# Effect of in situ acids removal on mixed glucose and xylose fermentation by *Clostridium tyrobutyricum*

**DOI:** 10.1186/s13568-015-0153-0

**Published:** 2015-10-29

**Authors:** George Nabin Baroi, Ioannis V. Skiadas, Peter Westermann, Hariklia N. Gavala

**Affiliations:** Section for Sustainable Biotechnology, Department of Chemistry and Bioscience, Aalborg University(AAU), A C Meyers Vænge 15, 2450 Copenhagen SV, Denmark; Department of Chemical and Biochemical Engineering, The Technical University of Denmark, Søltofts Plads 229, 2800 Kgs. Lyngby, Denmark

**Keywords:** Butyric acid, *Clostridium tyrobutyricum*, Fermentation, Inhibition, Reverse electro enhanced dialysis

## Abstract

In the present study, the effect of potassium ions and increasing concentrations of glucose and xylose on the growth of a strain of *Clostridium tyrobutyricum*, adapted to wheat straw hydrolysate, was investigated. Application of continuous fermentation of a mixture of glucose and xylose and in situ acid removal by reverse electro enhanced dialysis (REED) was investigated as a method to alleviate potassium and end-product inhibition and consequently enhance the sugar consumption rates and butyric acid productivity. It was found that glucose and xylose were not inhibitory up to a concentration of 50 and 37 g L^−1^ respectively, and that they were consumed at comparable rates when fermented alone. However, continuous fermentation of a mixture of glucose and xylose resulted in a significantly decreased xylose consumption rate compared to that of glucose alone, supporting the conclusion that *C. tyrobutyricum* has a lower affinity for xylose than for glucose. Potassium ions negatively affected the effective maximum growth rate of *C. tyrobutyricum* at concentrations higher than 5 g L^−1^ exhibiting a non-competitive type of inhibition. Continuous fermentation of a glucose and xylose mixture with simultaneous acid removal by REED resulted in a two to threefold increase of the glucose consumption rate, while the xylose consumption rate was enhanced sixfold compared to continuous fermentation without in situ acid removal. Similarly, butyric acid productivity was enhanced by a factor of 2–3, while the yield remained unaffected.

## Introduction

Butyric acid is currently produced from petroleum by oxidation of butyraldehyde obtained from oxosynthesis or hydroformylation of propylene (Playne 1985). The annual production of butyric acid accounts to 50,000 t (Sauer et al. 2008), which has numerous applications in the food, beverage, cosmetics and pharmaceutical industries (Zhang et al. 2009; Dwidar et al. 2012). Although butyric acid fermentation was discovered by Pasteur already in 1861 (Gottschalk 1985), it is only during the last decades that it has received increasing worldwide attention as the necessity for a bio-based and sustainable society has come in the forefront.

*Clostridium tyrobutyricum* has been extensively studied and it is characterized by high yields and selectivity for butyric acid with concurrent production of mainly acetic acid and hydrogen. It can also utilize both glucose and xylose as carbon source; two sugar monomers which are abundant in pretreated lignocellulosic material (Zhang et al. 2009; Dwidar et al. 2012). Thus, *C. tyrobutyricum* is a strong candidate for biological butyric acid production from 2nd generation biomasses. However, there are still challenges to overcome in this respect.

The specific growth rate of *C. tyrobutyricum* in glucose-based medium has previously been reported twofold higher than in a xylose-based medium (Liu and Yang 2006). Also, the growth rate differs when both glucose and xylose are present. In the study of Zhu et al. (Zhu et al. 2002) the specific growth rate of *C. tyrobutyricum* in a mixture of glucose and xylose (1:1) was half of the rate when only glucose was the carbon source. In a fed-batch experiment it was also observed that *C.tyrobutyricum* exposed to a glucose and xylose mixture followed a diauxic growth pattern, utilising preferably glucose and starting to utilize xylose only after all glucose was totally consumed. At subsequent feedings, parallel consumption of glucose and xylose was observed, however, the glucose consumption rate was threefold higher compared to that of xylose (Baroi et al. 2015a). Moreover, there were evidences that elevated concentrations of glucose affected the consumption rate of xylose negatively (Baroi et al. 2015b). A similar phenomenon has been observed with *Thermoanaerobacterium thermosaccharolyticum* W16 grown on a glucose and xylose mixture (Ren et al. 2008). Continuous processing, which allows for low concentrations of sugars in the reactor when a mixture of glucose and xylose is fermented could be advantageous in this respect. Continuous butyric acid fermentation with *C. tyrobutyricum* on glucose-based growth medium has been successfully carried out, especially when cell recycling was applied (Michel-Savin et al. 1990a, b; Du et al. 2012b).

Inhibition of microbial growth is another important factor that has to be taken into consideration. End product inhibiton is very common in anaerobic acid fermentations, for example in lactic acid (Iyer and Lee 1999) and propionic acid (Zhang et al. 1993) fermentations and has also been reported for butyric acid fermentation by *C.tyrobutyricum* (Michel-Savin et al. 1990a). Specifically, butyric acid has been reported to block the growth of *C. tyrobutyricum* at 40 g L^−1^, while even 10 g L^−1^ cause significant inhibition (Jiang et al. 2011). Zhou et al. (2014) observed that inhibition of *C. tyrobutyricum* growth started already at a butyric acid concentration between 3.6 and 7.2 g L^−1^. Further studies have also revealed that higher concentration of butyric acid negatively affects the activity of key metabolic enzymes, i.e. phosphotransacetylase, phosphotransbutyrylase and acetate kinase (Zhu and Yang 2004).

In-situ removal of the acids from the fermentation broth could be one of the solutions to reduce product inhibition (Zigova and Sturdik 2000). Using simultaneous solvent extraction, this technique was applied on butyric acid fermentation by *Clostridium butyricum* (Zigova et al. 1999). The problem with this technique is that an immiscible or partially immiscible organic solvent can be toxic to the bacteria. To minimize the toxic effect of the organic solvent, a fibrous bed bioreactor was developed successfully and used for butyric acid fermentation by *Clostridium tyrobutyricum* (Wu and Yang 2003). Application of in situ electrodialysis is an alternative method where no organic solvents are used and toxic effects are thus eliminated. This technique was tested for lactic (Boniardi et al. 1997), acetic, propionic (Zhang et al. 1993) and even for butyric acid extraction (Du et al. 2012a) and higher productivity was reported in all cases. Electrodialysis includes an anion exchange membrane (for acid–anion separation) and is subject to limitations by fouling effects. A relatively new technique, Reverse Electro Enhanced Dialysis, REED, (Rype and Jonsson 2002; Prado-Rubio et al. 2011) has been reported to be superior in that respect and it has so far been successfully applied to lactic acid extraction (Garde 2002) and recombinant protein production (Madsen et al. 2006). The REED system continuously removes acid anions from the fermentation broth by replacing them with hydroxide ions. This ion exchange also provides stabilization of the pH in the reactor. Thus, the usual practice of regulating the pH by adding a base (NaOH, KOH or NH_4_OH) can be avoided and inhibition from cations, especially when high amounts of acids are produced, is consequently prevented.

Besides end-product inhibition, addition of chemical bases for controlling the pH in acid fermentations, could inhibit microbial growth. In general, moderate concentrations of cations stimulate microbial growth while excessive amounts are inhibitory (Grady et al. 1999). Na^+^, K^+^ and NH_4_^+^ are the most common cations introduced in a fermentor during pH control and K^+^ has been reported to be the less inhibitory for yeasts since the cells already maintain a high intracellular potassium concentration (Casey et al. 2013). Moreover, it has been observed that xylose consumption in yeast is more adversely affected by the presence of cations than glucose consumption (Casey et al. 2013).

In the present study, the effect of K^+^ and increasing concentrations of glucose and xylose on the growth of *C. tyrobutyricum* was investigated. Application of continuous fermentation of a mixture of glucose and xylose and in situ acid removal by REED was investigated as a method to reduce potassium and end-product inhibition and consequently enhance the sugar consumption rates and butyric acid productivity.

## Materials and methods

### Microorganism and growth medium

*Clostridium tyrobutyricum,* strain DSMZ 2637, was obtained from Deutsche Sammlung von Microorganismen und Zellkulturen (DSMZ) and it was adapted to Pretreated and Hydrolyzed Wheat Straw (PHWS) by adaptive laboratory evolution technique as described in Baroi et al. (2015a). The adapted stain was stored at −80 °C in growth medium with 10 % glycerol and used throughout this study. The growth medium used has been described by O’brien and Morris (1971) and also applied in the studies of Baroi et al. (2015a, b). Experiments were carried out using either glucose or xylose alone or a mixture of both as carbon source.

### Analytical methods

Sugars were quantified by HPLC-RI as described in Baroi et al. (2015b). Acetic and butyric acids were quantified by gas gas chromatography with a flame ionization detector, FID and quantification of hydrogen gas was carried out by gas chromatography with a thermal conductivity detector, TCD as described in Baroi et al. (2015b). Total volatile suspended solids (VSS) were quantified according to standard methods (APHA 2005).

### Reverse electro enhanced dialysis—REED technology

The REED technology applied for in situ acid removal is a membrane separation process that combines elements from reversed electrodialysis and Donnan dialysis operations. Detailed descriptions of REED can be found in Garde (2002), Rype and Jonsson (2002) and Prado-Rubio et al. (2011). The REED membrane stack was built of 1 cell pair (915 cm^2^) and was equipped with anion-exchange (AX-REED) membranes to transport anions. The REED system was provided by Jurag Separation A/S (Denmark).

Acids separated by REED were collected as Na-salts. Dialysate and electrolyte were NaOH solutions at an initial concentration of 0.1 M. Fermentation broth and dialysate were recirculated at a flow rate of 400 and 200 mL min^−1^, respectively. Disinfection of the REED system and pipes was performed by circulating 400 ppm peracetic acid solution for 60–90 min followed by circulation of 10 l of sterile de-ionized (DI) water to wash out the disinfectant from the system. pH, maximum current and voltage were set for 7, 5A and 10 volt, respectively. The REED extraction efficiency was calculated as following (Eq. 1) (Baroi et al. 2015b):

1 $$REED\;extraction\;efficiency,\;\% = \frac{Butyric\;acid\;extracted}{Total\;butyric\;acid\;produced} \times 100$$

### Batch and continuous fermentations

**Fig. 1:**
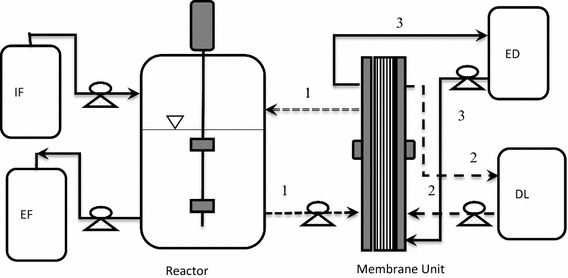
Schematic diagram of the experimental setup of continuous fermentation and in situ separation by REED. *IF* inflow, *EF* effluent, *ED* electrolyte, *DL* dialysate. *Lines 1*, *2* and *3* represent the fermentation broth, dialysate and electrolyte circulation, respectively (Baroi et al. 2015b)

Batch and continuous experiments were performed in a 3-L Applikon^®^ autoclavable glass reactor equipped with a controller for pH, temperature and agitation as described in Baroi et al. (2015b). The fermentation was carried out at 37 °C, 150 rpm and pH was maintained at 7 with 4M KOH when the REED system was not coupled to the fermentor. The fermentor was connected to the REED membrane unit as shown in Fig. 1, during experiments with in situ acid removal.

### Inhibition experiments and calculations

Three series of batch experiments were performed in order to investigate possible inhibition of the growth of *C. tyrobutyricum* from increasing concentrations of glucose, xylose and potassium ions.

Experiments with glucose as carbon source were carried out in triplicate in 117-ml serum vials sealed with rubber stoppers and aluminium crimps. The vials contained 50 ml of growth medium and the initial glucose concentration was in the range of 9–50 g L^−1^. The vials were inoculated with 10 % of a fully grown culture of *C. tyrobutyricum* on 5 g L^−1^ glucose. Glucose consumption was followed and initial glucose consumption rates were calculated.

Experiments with xylose as carbon source presented the challenge of a rather long and unpredictable lag phase after inoculation when the experiments were performed in serum vials, which made the calculation of initial rates difficult and non-accurate. Therefore, four xylose experiments were performed in the 3-L glass reactor with a pre-activation step of *C. tyrobutyricum* at a glucose and xylose mixture (1:1) with a total concentration of 2.25 g L^−1^. Subsequently and when the culture had reached the late exponential state it was spiked with a concentrated xylose solution to establish an initial xylose concentration in the range of 5–40 g L^−1^. Xylose consumption was followed and initial xylose consumption rates were calculated.

The set-up of the experiments with glucose and xylose allowed for the same initial concentration of microbial cells in all experiments with the same carbon source. Considering Monod kinetics (Eq. 2), comparison of initial rates for the carbon source consumption allowed for direct deduction of the effective maximum specific growth rate µ_max,eff_, in each case, as the substrate consumption rate becomes zero-order (Eq. 3) when K_s_ ≪ S. Therefore, comparison of initial rates under the conditions mentioned before, allows also for drawing conclusions on the existence or not of substrate inhibition, as substrate inhibition negatively affects the effective maximum growth rate (Rittmann and McCarty 2001).

2 $$\left( {\frac{dS}{dt}} \right)_{eff,in} = - \frac{1}{{Y_{X/S}}} \cdot \frac{{\mu_{{\rm max}, eff}\cdot S_{in} }}{{K_{S,eff} + S_{in} }} \cdot X_{in}$$

where S_in_ and X_in_ is the initial substrate and microbial biomass concentration respectively, µ_max, eff_ is the effective maximum growth rate, K_S,eff_ is the effective saturation constant and Y_X/S_ is the microbial cell yield.

3 $$\left( {\frac{dS}{dt}} \right)_{eff,in} = - \frac{1}{{Y_{X/S}}} \cdot \mu_{{\rm max} ,eff} \cdot X_{in}$$

Four batch experiments with potassium ions concentration of 5, 10, 15 and 20 g L^−1^ were performed in the 3-L glass reactor. Potassium ions were supplied in the form of the K_2_HPO_4_ and KH_2_PO_4_ buffering system, while the concentration of other nutrients in the growth medium was as described previously. A mixture of glucose and xylose at a mass ratio of 1.3:1 was used as carbon source at a low initial concentration (<4 g L^−1^) in order to ensure a neutral pH in all experiments. The growth of the microbial cells was monitored by measuring the optical density, OD, at 660 nm. Microbial biomass concentrations were calculated in g L^−1^ using Eq. 4, which represents a calibration curve of OD_660_ versus Volatile Suspended Solids (VSS) obtained for *C. tyrobutyricum* grown on a 5 g L^−1^ glucose and xylose-based medium.

4 $$VSS = 0.5251 \cdot OD_{660} - 0.0235$$

Estimation of the µ_max,eff_ was performed for each experiment at the early exponential phase where K_S_ ≪ S by integrating Eqs. 5 and 6. The yields of microbial biomass were calculated based on the experimental measurements at the specific time period according to Eq. 7.

5 $$\left( {\frac{dX}{dt}} \right)_{eff} = \mu_{{\rm max} ,eff} \cdot X$$

6 $$\left( {\frac{dS}{dt}} \right)_{eff} = - \frac{1}{{Y_{{X/S}}}} \cdot \mu_{ {\rm max} ,eff} \cdot X$$

7 $$Y_{X/S} = \frac{\Delta X}{\Delta S}$$

where S and X is the substrate and microbial biomass concentration respectively, µ_max, eff_ is the effective maximum growth rate, and Y_X/S_ is the yield of the microbial cells.

## Results

### Inhibition experiments

Initial consumption rates of glucose and xylose in the batch experiments with different initial glucose and xylose concentration are shown in Table 1.

**Table 1:** Initial glucose and xylose consumption rates at different sugar initial concentration

Initial concentration (g L^−1^)	Initial sugar consumption rate (g L^−1^ h^−1^)
Glucose, 8.9	0.33 ± 0.09
Glucose, 16.8	0.28 ± 0.06
Glucose, 26.0	0.27 ± 0.04
Glucose, 34.8	0.39 ± 0.14
Glucose, 50.7	0.27 ± 0.05
Xylose, 6.2	0.25
Xylose, 12.9	0.39
Xylose, 26.7	0.18
Xylose, 37.0	0.33

**Fig. 2:**
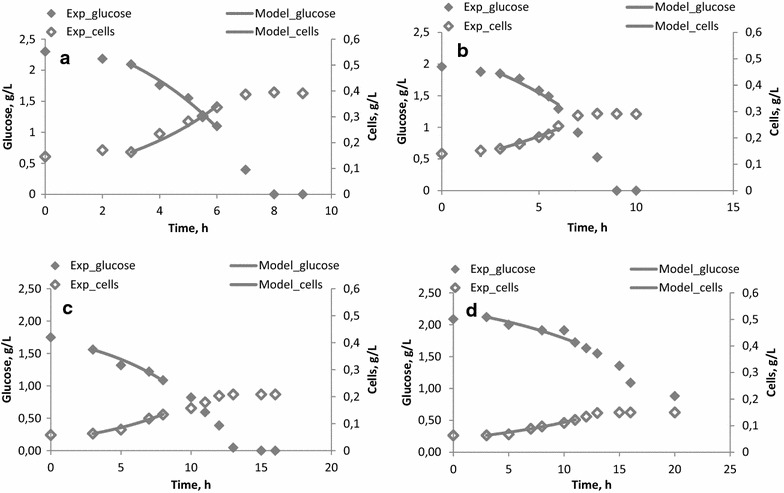
Experimental and theoretical (model) glucose and microbial cells concentration profiles during experiments with 5 (**a**), 10 (**b**), 15 (**c**) and 20 (**d**) g L^−1^ K^+^. Theoretical simulations are shown with *solid lines*

During the batch experiments with different concentrations of potassium ions added, xylose consumption was negligible and therefore calculations were only based on glucose uptake. Experimental and theoretical (model) glucose and microbial cell concentration profiles are shown in Fig. 2. Table 2 shows the experimental values for the yield of microbial cells and calculated values for the effective maximum growth rate.

**Table 2:** Yields of microbial cells and effective maximum growth rates during the experiments with increasing concentration of K^+^

Concentration of K^+^ (g L^−1^)	Microbial biomass yield (g g^−1^)	Effective maximum growth rate (h^−1^)
5	0.175	0.246
10	0.155	0.130
15	0.149	0.152
20	0.147	0.081

### Continuous fermentations without acid removal

Two continuous fermentation experiments were performed at 1 d Hydraulic Retention Time (HRT). The first (C-1) was fed with an influent of 50 g L^−1^ glucose, while the second (C-2) was fed with a mixture of glucose (38 g L^−1^) and xylose (22 g L^−1^). The fermentor was initially operated in batch mode until xylose was almost totally consumed and subsequently the operation was shifted to continuous mode. Glucose and xylose concentrations and consumptions rates, hydrogen, butyric and acetic acid production rates and yields obtained at each steady state are shown in Table 3.

**Table 3:** Characteristics of the steady states during continuous fermentations with glucose (C-1) and a mixture of glucose and xylose (C-2) as carbon source

	C-1	C-2
Operating features		
HRT (d)	1	1
Influent glucose concentration (g L^−1^)	50	38
Influent xylose concentration (g L^−1^)	–	22
Steady state characteristics		
Glucose concentration (g L^−1^)	29.19	4.77
Xylose concentration (g L^−1^)	–	17.87
Butyric acid concentration (g L^−1^)	6.41	9.94
Acetic acid concentration (g L^−1^)	2.58	3.12
Glucose consumption rate (g L^−1^ h^−1^)	0.72	0.85
Xylose consumption rate (g L^−1^ h^−1^)	–	0.11
Acetic acid production rate (g L^−1^ h^−1^)	0.10	0.12
Acetic acid yield (g g^−1^ sugars)	0.145	0.13
Butyric acid production rate (g L^−1^ h^−1^)	0.26	0.37
Butyric acid yield (g g^−1^ sugars)	0.36	0.39
Butyric acid selectivity (g g^−1^ acids)	0.76	0.76
Hydrogen production rate (L L^−1^ h^−1^)	0.164	0.23

### Continuous fermentations with in situ acid removal by REED

Four continuous fermentation experiments were performed with the REED system connected to the fermentor to allow in situ acid-removal during the fermentation. The operating features of the experiments were as follows:

REED-1: 50 g L^−1^ influent glucose at 1 d HRT
REED-2: 38 g L^−1^ influent glucose and 22 g L^−1^ influent xylose at 1 d HRT
REED-3: 54 g L^−1^ influent glucose and 34 g L^−1^ influent xylose at 1 d HRT
REED-4: 54 g L^−1^ influent glucose and 34 g L^−1^ influent xylose at 2 d HRT

Results obtained from REED-1 were directly comparable to the continuous experiment with 50 g L^−1^ influent glucose concentration (C-1, Table 3). Similarly, results of REED-2 were comparable to the continuous experiment with glucose and xylose as carbon source (C-2, Table 3). REED-3 focused on the effect of increased influent concentration compared to REED-2, while REED-4, which was operated at lower dilution rate was directly comparable to REED-3.

The continuous fermentation was performed after a batch activation phase as described before. The REED system was connected as soon as the continuous operation mode was applied. Glucose and xylose concentrations and consumptions rates, hydrogen, butyric and acetic acid production rates and yields obtained at each steady state are shown in Table 4.

**Table 4:** Characteristics of the steady states during continuous fermentation experiments with in situ acids removal by REED

	REED-1	REED-2	REED-3	REED-4
Operating features				
HRT (d)	1	1	1	2
Influent glucose concentration (g L^−1^)	50	38	54	54
Influent xylose concentration (g L^−1^)		22	34	34
Steady state characteristics				
Glucose concentration (g L^−1^)	<0.45	<0.45	15.16	<0.45
Xylose concentration (g L^−1^)	–	9.62	20.16	3.93
Butyric acid concentration (g L^−1^)	5.21	4.08	2.76	2.79
Acetic acid concentration (g L^−1^)	1.25	1.73	0.93	0.95
Glucose consumption rate (g L^−1^ h^−1^)	^1^2.08	^1^1.64	1.81	1.17^a^
Xylose consumption rate (g L^−1^ h^−1^)	–	0.65	0.72	0.65
Acetic acid production rate (g L^−1^ h^−1^)	0.23	0.39	0.45	0.27
Acetic acid yield (g g^−1^ sugars)	0.11	0.17	0.18	0.15
Butyric acid production rate (g L^−1^ h^−1^)	0.79	0.78	0.85	0.55
Butyric acid yield (g g^−1^ sugars)	0.38	0.34	0.34	0.30
Butyric acid selectivity (g g^−1^ acids)	0.78	0.67	0.65	0.67
Hydrogen production rate (L L^−1^ h^−1^)	0.50	0.51	0.62	NM^b^
REED extraction efficiency (%)	72.2	84.59	89.86	93.3

^a^Highest possible rate achieved (kinetically non-limited)

^b^Not measured

## Discussion

### Inhibition experiments

The consumption rates of glucose and xylose did not exhibit any decreasing trend with increasing sugar concentration (Table 1), implying that neither glucose nor xylose inhibited *C. tyrobutyricum* growth at the concentration range tested, namely up to a concentration of 50 and 37 g L^−1^ respectively. The average value for initial rates were 0.31 ± 0.05 and 0.29 ± 0.09 g L^−1^ h^−1^ for glucose and xylose consumption, respectively, demonstrating that the consumption rates of glucose and xylose were very similar when sugars were fermented alone. However, xylose was consumed at a eightfold lower rate than glucose during continuous fermentation of a mixture of these sugars (Table 3, experiment C-2) and moreover the same observation had been made during batch fermentations of mixture of glucose and xylose even at a low initial concentration (<4 g L^−1^) (Baroi et al. 2015a). These results support the previously made hypothesis (Baroi et al. 2015a) that *C. tyrobutyricum* has a lower affinity for xylose than for glucose.

On the other hand, increasing concentrations of potassium ions negatively affected the effective maximum growth rate (Table 2). According to Rittmann and McCarty (2001) this implies that potassium ions exhibit a non-competitive type of inhibition to the growth of *C. tyrobutyricum*. It is hereby worthy to mention that although inhibition from organic acids has been studied a lot in the past, also for the production of butyric acid from *C. tyrobutyricum* (Michel-Savin et al. 1990a; Jiang et al. 2011; Zhou et al. 2014; Zhu and Yang 2004), no attention has been given to the inhibition due to the cations concentration, which seems to also significantly affect the growth of *C. tyrobutyricum*. Based on the obtained results and since more often than not, controlling the pH during a fermentation process may result in cations accumulation in considerable concentrations, any possible inhibition effects should be considered and addressed.

### Continuous fermentations without acid removal

Glucose consumption rates were higher than the average rates calculated from the batch experiments, despite that the acid concentration had reached inhibitory levels (Jiang et al. 2011; Zhou et al. 2014), especially in experiment C-2. As mentioned before, it is also noticeable that the xylose consumption rate was almost eightfold lower than that of glucose when a mixture of glucose and xylose was fermented (experiment C-2) although glucose and xylose exhibited comparable consumption rates when fermented alone. The same observation was made during batch fermentation of a mixture of glucose and xylose and was attributed to the higher affinity of *C. tyrobutyricum* for glucose than xylose (Baroi et al. 2015a). Potassium ions concentration was calculated to be approximately 4 and 6.4 g L^−1^ for C-1 and C-2, respectively. This means that potassium inhibition will also occur for higher influent sugar concentrations and consequently higher acid concentration in the fermentor, suppressing the growth of *C. tyrobutyricum* even more and consequently the rate of the fermentation and butyric acid productivity. Therefore, reverse electro-enhanced dialysis was investigated as a method for in situ acid removal and neutralization of the fermentation broth without addition of potassium ions or other cations eliminating the two major sources of inhibition: organic acids and cations.

### Continuous fermentations with in situ acid removal by REED

**Fig. 3:**
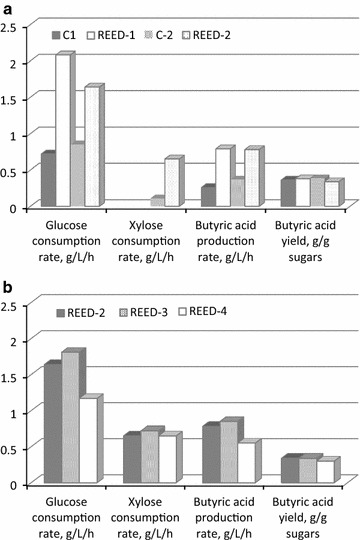
Glucose and xylose consumption rates and butyric acid production rate and yield obtained in the continuous experiments of **a** glucose (C-1 and REED-1) and mixture of glucose and xylose (C-2 and REED-2) with and without in situ acid removal by REED and **b** mixture of glucose and xylose with in situ acid removal by REED

In Fig. 3 direct comparisons of glucose and xylose consumption rates and butyric acid productivity and yield during continuous fermentations with and without REED are shown.

During the experiments with REED, an impressive increase in the consumption rates of both glucose and xylose was achieved. Specifically, glucose consumption rate increased two to threefold, while the consumption rate of xylose exhibited a sixfold increase (0.65 compared to 0.11 g L^−1^ h^−1^) when a mixture of glucose and xylose was fermented. The positive effect of the application of the REED system was more pronounced in the case of xylose consumption rates implying that organic acids and/or cations might exhibit a stronger inhibition on xylose consumption. The same observation has been made by Casey et al. (2013) when glucose and xylose were fermented by yeast cells in the presence of salts and the underlying mechanisms would be worthy of further investigation. Butyric acid productivity was also enhanced by 2 to threefold, while the yield was similar with and without the REED system (0.37 compared to 0.36 in average). Increase in the influent sugars concentration (REED-3) did not cause any significant change in rates and yield (Fig. 3b), however, the level of residual, non-fermented sugars increased significantly (Table 4). Doubling of the HRT (REED-4) resulted in lowering of the concentration of glucose and xylose and as anticipated, such a decrease in dilution rate affected the productivity of butyric acid negatively (0.55 compared to 0.85 g L^−1^ h^−1^ in REED-4 and REED-3, respectively). In overall, reverse electro enhanced dialysis (REED) was successfully applied as a method to alleviate potassium and end-product inhibition during butyric acid fermentation of a mixture of glucose and xylose by *Clostridium tyrobutyricum*. It was shown that REED enhanced the xylose and glucose consumption rate with the effect being more pronounced in the case of xylose (sixfold compared to a threefold increase for glucose). Butyric acid productivity was also increased by a factor of 2–3 while the yield remained almost unaffected. Thus, in situ acid removal by REED is a promising technology for not only enhancing sugar consumption rates and butyric acid productivity but also for securing simultaneous consumption of both glucose and xylose. This is very important especially when bioprocessing of second generation biomasses is targeted since xylose constitutes a significant fraction of the sugars content.

**Fig. 4:**
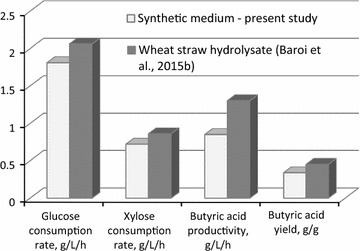
Glucose and xylose consumption rates and butyric acid production rate and yield obtained in the continuous experiments with wheat straw hydrolysate and synthetic medium at 1 d HRT and in situ acid removal by REED with an influent sugar concentration of 54–55 and 34–35 g L^−1^ glucose and xylose, respectively

The *C. tyrobutyricum* strain used in this study was already adapted to wheat straw hydrolysate as previously mentioned by applying the adaptive laboratory evolution technique (Baroi et al. 2015a). It is noticeable and hereby confirmed that its performance in regards to glucose and xylose consumption rates and butyric acid productivity was superior when wheat straw hydrolysate was used (Baroi et al. 2015b) compared to the synthetic medium used in the present study. Glucose and xylose consumptions rates and butyric acid production rates and yields obtained with wheat straw hydrolysate and synthetic medium at 1 d HRT with in situ acid removal by REED are shown in Fig. 4. Wheat straw hydrolysate contained 55 and 35 g L^−1^ glucose and xylose, respectively, so the results obtained were directly comparable to the results of the experiment REED-3 of the present study (with 54 and 34 g L^−1^ influent glucose and xylose, respectively). The *C. tyrobutyricum* strain that was previously adapted to wheat straw hydrolysate exhibited a 14–20 % enhanced glucose and xylose consumption rate, a 32 % higher butyric acid yield and an overall 53 % increased butyric acid productivity compared to those obtained when synthetic growth medium was used. Therefore, adaptive laboratory evolution technique, which was applied on *C. tyrobutyricum* strain, resulted also in higher efficiencies from a process point of view besides its usefulness in overcoming inhibition/toxicity.
